# From colonization to invasion: genomic and phenotypic comparison of faecal and bloodstream isolates from the same patients

**DOI:** 10.1099/jmm.0.002147

**Published:** 2026-04-08

**Authors:** Aakash Khanijau, Ellie Allman, Ralfh Pulmones, Richard N. Goodman, Daire Cantillon, Rachel McGalliard, Christopher M. Parry, Enitan D. Carrol, Adam P. Roberts

**Affiliations:** 1 Department of Tropical Disease Biology, Liverpool School of Tropical Medicine, Pembroke Place, Liverpool L3 5QA, UK; 2 Institute of Infection, Veterinary and Ecological Sciences, University of Liverpool, Liverpool, UK; 3 Alder Hey Children’s NHS Foundation Trust, Liverpool, UK; 4 Centre for Tropical Medicine and Global Health, Nuffield Department of Medicine, University of Oxford, Oxford, UK

**Keywords:** antimicrobial resistance (AMR), biofilm, bloodstream infection, *Escherichia coli*, *Klebsiella*, plasmids

## Abstract

**Introduction.** Gram-negative bloodstream infections (GNBSIs) carry a significant global health burden. *Escherichia coli* and *Klebsiella pneumoniae* are the two most common causes of healthcare-associated GNBSI, which may arise from gastrointestinal tract (GIT) colonization.

**Gap Statement.** We do not fully understand how GNBSIs arise from GIT colonization.

**Aim.** To understand *E. coli* and *K. pneumoniae* genomic and phenotypic adaptations that underpin transition from GIT colonization to invasive bloodstream infection.

**Methodology.** This study identified ‘linked’ faecal and blood isolates from children with healthcare-associated GNBSI caused by *E. coli* and *K. pneumoniae*. Linked pairs were compared for antimicrobial resistance and biofilm formation and underwent comparative genomic analysis via whole-genome sequencing, comparative average nucleotide identity and core genome single nucleotide polymorphism (SNP) analysis.

**Results.** Five isolate pairs (three *E. coli*, two *K. pneumoniae*) showed high relatedness, supporting the GIT origin of bloodstream infection. Isolates within pairs had identical virulence genes, whereas phenotypic assays revealed changes in antimicrobial susceptibility, with one pair undergoing changes in resistance gene profiles and increased biofilm formation in four out of five isolates.

**Conclusion.** This study provides insight into within-host evolution from gastrointestinal colonization to bloodstream invasion in Gram-negative pathogens. Convergence on metabolic adaptation and biofilm formation suggests that these traits may be advantageous in healthcare-associated GNBSI. Further studies involving larger cohorts alongside functional validation of mutations are needed to better understand GNBSI pathogenesis.

## Data Summary

The BioProject number for this study is PRJNA1295786, which contains the ten accession numbers for the individual genomes SRX29836134–SRX29836143.

## Introduction

Gram-negative bloodstream infections (GNBSIs) contribute significantly to the global burden of paediatric sepsis, accounting for 30–50% of all bloodstream infections worldwide [1,2]. Healthcare-associated bloodstream infections, such as central line-associated bloodstream infections (CLABSIs), often affect neonates or other critically ill children and are associated with significant mortality [3].

The two most clinically significant organisms implicated in GNBSI are *Klebsiella pneumoniae* and *Escherichia coli* [4,5]. The emergence of antimicrobial resistance (AMR) in these pathogens presents a significant challenge. Particularly, extended-spectrum beta-lactamase (ESBL)-producing isolates exhibit resistance to third-generation cephalosporins and beta-lactam/beta-lactamase inhibitor combinations. As such, the World Health Organization has designated ESBL-producing *K. pneumoniae* and *E. coli* as priority pathogens for which new antibiotics are required [6].

Both *K. pneumoniae* and *E. coli* are persistent colonizers of the human gastrointestinal tract (GIT), which is believed to be an important reservoir for subsequent GNBSI [7]. The transition from GIT colonization to GNBSI can occur via invasion of other body sites or medical devices, leading to secondary bloodstream infection, or direct translocation across the gut mucosa, causing primary bloodstream infection. The latter is implicated in 20–50% of cases of healthcare-associated infections [7]. The transition from GIT colonization to bloodstream invasion depends on host, clinical and environmental influences, combined with specific pathogen characteristics. Host factors that can predispose to GIT-bloodstream transition include immunosuppression, critical illness and antimicrobial exposure [7,8]. Pathogen factors include advantageous phenotypes such as AMR, bloodstream fitness and biofilm formation, which may allow invasion, survival and dissemination in the bloodstream. Biofilm formation is of particular interest in the context of CLABSI, as growth within a biofilm provides protection against host defence and antimicrobial agents and may act as a nidus for ongoing bloodstream dissemination.

Many antimicrobial resistance genes (ARGs) and virulence genes underpinning AMR and invasive infection, respectively, are well characterized. However, predisposing genotypes and specific genetic changes undergone within-host that may underpin GIT–blood transition are seldom studied, perhaps due to the scarcity of such paired isolates from the same patient. This includes genomic adaptations like acquisition or loss of virulence genes and plasmids, and regulatory or metabolic alterations. Furthermore, the relationship between many genomic adaptations and advantageous phenotypic changes remains unclear, as well as the relationship between AMR and invasive infection. Combined phenotypic and genomic comparison of faecal and bloodstream isolates, obtained from the same patient, could improve understanding of intra-host evolution involved in the pathogenesis of GNBSI and translate to informing intervention strategies.

In this study, we aimed to identify genomically ‘linked’ pairs of faecal and blood Gram-negative isolates, which were likely to have undergone GIT-blood transition and evolution within patients to cause GNBSI. For each ‘linked’ pair, we investigated if there had been any loss or acquisition of virulence genes, ARGs and plasmids between faecal isolates and their blood counterpart. We then investigated if there were any differences in two phenotypes of interest: AMR and biofilm formation. Finally, we used a comparative genomic approach to identify further genomic changes that could explain any observed phenotypic differences or otherwise contribute to the transition from GIT colonization to bloodstream invasion.

## Methods

### Clinical isolates, clinical data collection and ethics

A library of 81 stored bacterial isolates from Alder Hey Children’s Hospital, Liverpool, UK, was interrogated for pairs of ESBL-producing *E. coli* and *K. pneumoniae* isolates from the same patient, where the same pathogen had been identified on a routine faecal surveillance culture and in a subsequent blood culture. Isolates were stored in LB (Luria-Bertani) broth (Sigma-Aldrich) and 20% glycerol at −80 °C. Anonymized clinical information was obtained from patient records, including demographics, source of GNBSI and antimicrobial exposure between isolate dates. Ethical approval for linking anonymized data was granted by the University of Liverpool as part of a separate study for which the isolate library was created (REC reference 255669).

### Whole-genome sequencing and comparative genomics

Short-read whole-genome sequencing (WGS) and assembly were performed at the Centre of Genomic Research (University of Liverpool) using the Illumina NovaSeq SP (2×150 bp). Raw reads were trimmed for adapter sequences using Cutadapt v1.2.1 [9]. Reads were then quality-trimmed using Sickle v1.200 (https://github.com/najoshi/sickle) with a minimum window quality score of Q20, and reads shorter than 20 bp after trimming were discarded. Trimmed reads were assembled using SPAdes v3.13.1 [10] with default parameters. Contigs were filtered to remove any shorter than 200 bp in length. Assembled genomes were analysed for the following characteristics, which were compared between faecal and blood isolates of each patient pair. Core genome multi-locus sequence typing (cgMLST) was performed using Kleborate [11] for *K. pneumoniae* isolates and using the Institut de Pasteur database (https://bigsdb.pasteur.fr/about/) for *E. coli* isolates. Kaptive [12] was used to identify wzi-type, K-type and O-type of *K. pneumoniae* isolates, and SeroTyper 2.0 and CHtype to determine the H-type, O-type and FumC-type of *E. coli* isolates.

To assess relatedness further, average nucleotide identity (ANI) was then compared between isolates using OrthoANI [13]. Following this, the number of core genome single nucleotide polymorphisms (SNPs) between faecal and blood isolates of each pair was calculated. SNPs were predicted using Snippy v.4.3.6 (https://github.com/tseemann/snippy) by aligning raw reads of assemblies to the reference genomes MG1655 and HS11286, for *E. coli* and *K. pneumoniae*, respectively. Core genome SNPs were extracted with Snippy-core, and intra-species SNPs were plotted and counted in R v.4.3.1 [14]. ResFinder 4.2.2 [15] was used to determine the presence of ARGs and provide a genotypic prediction of resistance for each isolate. VF analyser (http://www.mgc.ac.cn/VFs/) was used to identify virulence genes in each isolate. PlasmidFinder [16] was used to identify the presence of plasmids. Isolate genomes were annotated using the RAST annotation tool [17], and pairs underwent comparative genomic analysis using Breseq version 0.38.3 [18] using default parameters. Mutations between blood isolates and the putative counterpart faecal ancestor were categorized as insertions/deletions (indels), non-synonymous SNPs (nsSNPs), synonymous SNPs (sSNPs) or intergenic SNPs (iSNPs). Mutations within or <100 bp upstream of a gene were classified into Cluster of Orthologous Groups (COG) functional categories using EGGNOG 5.0. Mutations involving hypothetical proteins were classified as ‘Function unknown’, and mutations without genes within 100 bp were ‘Unclassified.’ Any mutations relating to virulence genes, ARGs or previously described bloodstream survival factors were identified. In addition, mutations in genes previously reported to affect AMR or biofilm formation were identified.

### Antimicrobial susceptibility testing

Six antimicrobials from the contemporaneous local prescribing guidance for Gram-negative infections were used for antimicrobial susceptibility testing (AST): cefotaxime, gentamicin, ciprofloxacin, amoxicillin-clavulanic acid (co-amoxiclav), piperacillin-tazobactam and meropenem. Kirby–Bauer disc diffusion testing was used for initial AST following EUCAST guidelines [19]. Zone sizes were interpreted as per the EUCAST clinical breakpoints, to give a phenotype of sensitive (S), resistant (R) or in the ‘Area of Technical Uncertainty’ [20]. Three technical and three biological repeats were performed, and the mode was recorded as the phenotype for each isolate and compared within each pair. In addition, the mean zone size, along with sem, was recorded for each isolate, and the Mann–Whitney U test was used to compare differences between faecal and blood isolates for each patient pair. Any identified differences between faecal and blood isolates in each patient pair were confirmed using broth microdilution to quantify the fold change in MIC. Broth microdilution was performed following EUCAST guidelines [21] and with three biological and three technical replicates.

### Biofilm formation

Crystal violet assay was performed, derived from two previously published protocols [22,23]. Isolates were incubated at 37 °C on LB agar for 18 h. A single colony of each isolate was transferred to 10 ml of Mueller–Hinton broth 2 (MHB2; Sigma-Aldrich) and incubated at 37 °C at 200 r.p.m. for a further 18 h. Cultures were diluted to an OD_600_ of 0.01 in fresh MHB2. One hundred and fifty microlitres of each isolate were transferred in a U-bottom plate, with 150 µl sterile MHB2 used as a negative control. After 24 h incubation, the biomass was transferred to a flat-well plate, which was used to measure OD_600_. The remaining biofilm in the U-bottom plate wells was stained with 1% crystal violet and subsequently solubilized with 30% acetic acid. The final OD_600_ was measured in a flat-well plate. This value was then normalized to the initial OD_600_ of the biomass to quantify biofilm formation. The assay was performed as three biological replicates and four technical replicates. Mean OD_600_ of blood and faecal isolates was compared using a paired t-test, and sem was calculated.

### Statistical analysis and data visualization

All statistical analysis and data visualization were performed using ggplot2 in R version 4.3.2 [14].

## Results

### Clinical isolates and clinical context of GNBSI

Five pairs of isolates, two *K. pneumoniae* and three *E. coli*, were identified for use in this study (Table 1). Time between faecal (first sample) and blood sample isolation ranged from 17 to 144 days. Patients from whom isolates were derived were aged 1 month to 12 years. All five cases of GNBSI were diagnosed as CLABSI, and four out of five cases occurred in the intensive care unit (ICU). Patients received between two and six different antimicrobials between faecal and blood sample isolation.

**Table 1.** Characteristics of isolate pairs and clinical data of patients with GNBSI

Pair	Species of isolate pair	Days from faeces – blood isolation	Age at GNBSI	CLABSI	ICU patient at time of GNBSI	Antibiotic exposure between faeces and blood isolate
1	*K. pneumoniae*	144	12 years	Y	N	Ciprofloxacin, clindamycin, ceftriaxone, piperacillin-tazobactam, co-trimoxazole, gentamicin
2	*K. pneumoniae*	87	11 months	Y	Y	Ciprofloxacin, cefalexin, piperacillin-tazobactam, ceftriaxone, vancomycin, gentamicin
3	*E. coli*	185	9 months	Y	Y	Co-amoxiclav, cefalexin
4	*E. coli*	31	12 months	Y	Y	Ciprofloxacin, meropenem
5	*E. coli*	17	1 month	Y	Y	Ciprofloxacin, vancomycin

### Genomic relatedness of faecal–blood isolate pairs

Genome sequencing statistics are detailed in Table S1, available in the online version of this article. To initially assess relatedness between faecal and blood isolates, cgMLST was performed. In five out of five pairs, faecal and blood isolates shared the same sequence type (Table 2). In Pair 1, *K. pneumoniae* isolates were from the sequence type ST922, and in Pair 2, *K*. *pneumoniae* isolates were from the sequence type ST461 (Table 2). In Pairs 3 and 4, *E. coli* isolates were from the globally dominant sequence type ST131, whereas in Pair 5, isolates were from sequence type 12 (Table 3). *K. pneumoniae* isolates were then compared for K-type, O-type and wzi-type, which were concordant between faecal and blood isolates in both pairs. *E. coli* isolates were compared for O-type, H-type and FumC-type, and again, all were concordant within each pair.

**Table 2.** MLST, K-type, O-antigen type and wzi-type of *K. pneumoniae* isolates within pairs, determined using Kleborate and Kaptive

Species	*K. pneumoniae*			
Pair	Pair 1		Pair 2	
Isolate	Faeces	Blood	Faeces	Blood
MLST	ST922	ST922	ST461	ST461
K-type	K28	K28	K25	K25
O-type	O1	O1	O1	O1
wzi-type	wzi84	wzi84	wzi25	wzi25

**Table 3.** Phylotype and MLST of *E. coli* isolates within pairs using ClermonTyping and Institut de Pasteur database

Species	*E. coli*					
Pair	Pair 3		Pair 4		Pair 5	
Isolate	Faeces	Blood	Faeces	Blood	Faeces	Blood
MLST	ST131	ST131	ST131	ST131	ST12	ST12
FumC-type	C40	C40	C40	C40	C13	C13
O-type	H4	H4	H4	H4	H1	H1
H-type	O25	O25	O25	O25	O4	O4

**Fig. 1.**
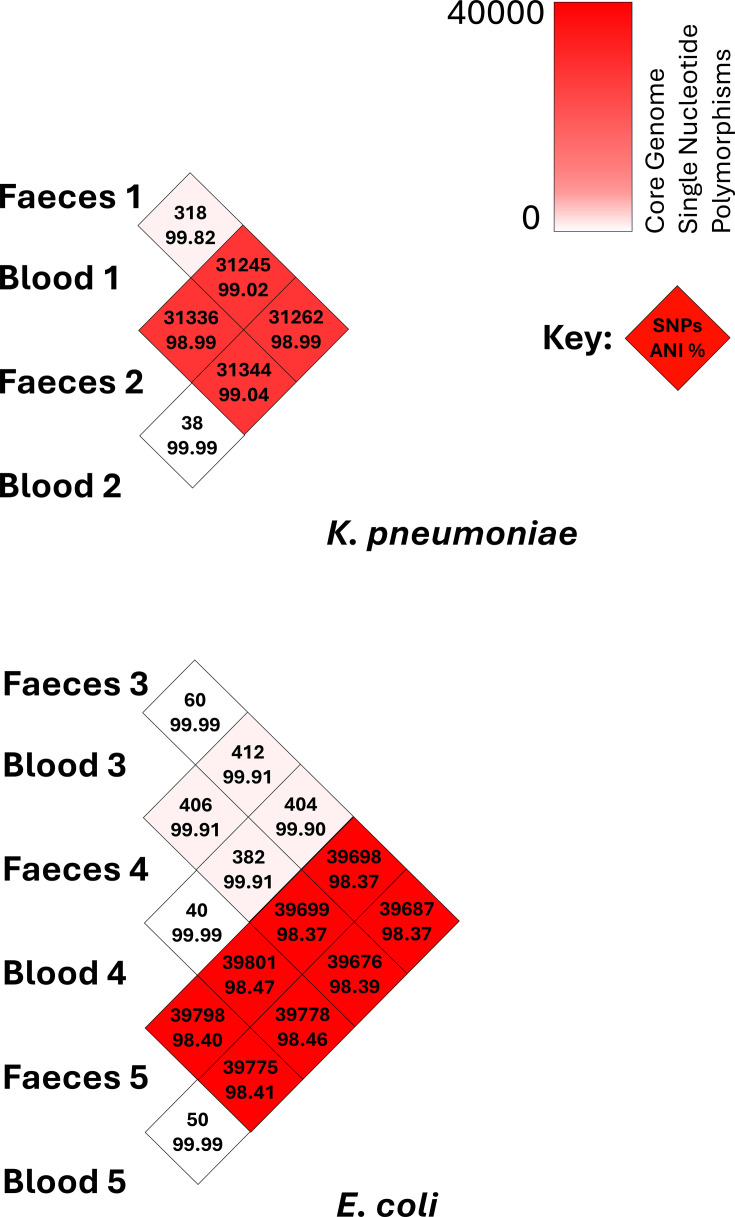
Pairwise comparison of ANI and core genome SNPs between isolates.

Next, relatedness was further assessed using ANI (Fig. 1). In 4 out of 5 pairs (2, 3, 4 and 5), the faecal–blood isolates shared an ANI of 99.99%, whereas isolates in Pair 1 shared an ANI of 99.82%. Within a comparison between all isolates across all patient pairs, each faecal isolate was most closely related to its corresponding blood isolate from the same patient. Given these findings, all pairs were at this point considered to be genomically ‘linked’ and included for further study.

Isolates were next compared for core genome SNP differences (Fig. 1 and Table S2). For *K. pneumoniae* isolates, Pair 2 exhibited the lowest SNP difference (38 SNPs), whilst Pair 1 showed the highest (318 SNPs). In *E. coli* isolates, within-pair SNP distances ranged from 40 SNPs in Pair 4 to 60 SNPs in Pair 3. These findings were in alignment with the ANI comparison.

### Initial genomic comparison: ARGs, virulence genes and plasmids

**Fig. 2.**
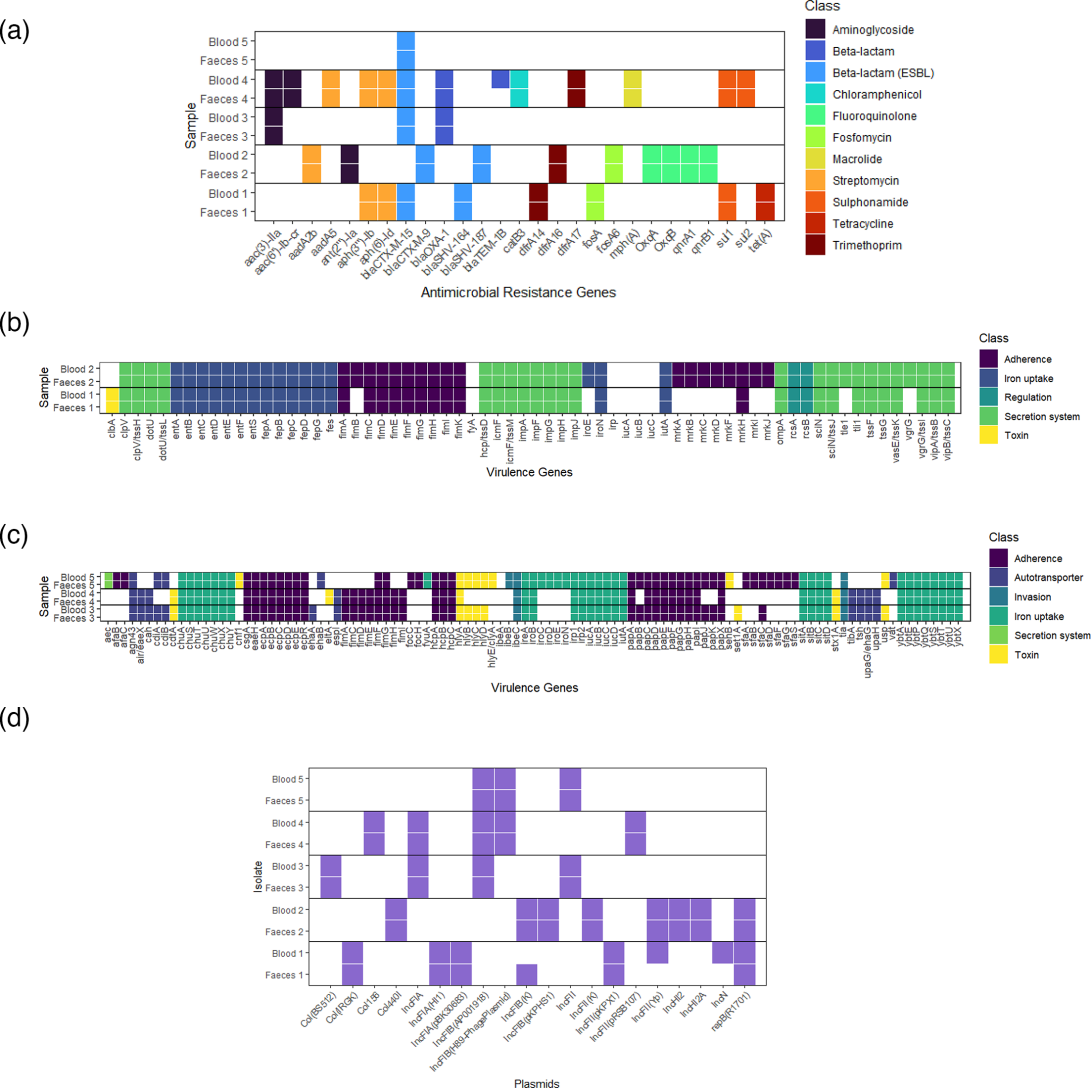
(a) ARG presence in all isolates. Each tile represents the presence of an ARG as detected by ResFinder 4.2.2. using WGS data. ARGs are categorized by drug class that they are predicted to confer resistance to. (b) Virulence gene presence in *K. pneumoniae* isolates. Each tile demonstrates the presence of a virulence gene as detected by the VF analyser using WGS data. Virulence genes are categorized by class of virulence factor encoded by the gene. (c) Virulence gene presence in *E. coli* isolates. Each tile demonstrates the presence of a virulence gene as detected by the VF analyser using WGS data. Virulence genes are categorized by class of virulence factor encoded by the gene. (d) Plasmid presence in all pairs of isolates. Each tile demonstrates the presence of a plasmid replicon gene in each isolate as detected by PlasmidFinder, using WGS data.

At this point, all pairs were considered to be ‘linked’, and an initial genomic comparison of ARGs, virulence genes and plasmids of the faecal and blood isolates within each pair was performed (Fig. 2). There were between 1 and 13 ARGs detected in each isolate, predicted to confer resistance to various drug classes (Fig. 2a). The presence of at least one ESBL gene was confirmed in each isolate. ARG profiles were identical within pairs, except for Pair 4, in which *bla*TEM-1B was detected in the blood isolate but not the faecal counterpart, suggesting acquisition between the dates of sampling, possibly during GIT–blood translocation.

There were between 51 and 86 virulence genes detected in each isolate, encoding multiple classes of virulence factors (Fig. 2b, c). Virulence gene presence was identical between faecal and blood isolates, indicating no loss or acquisition of virulence genes in GIT–blood transition in any pair.

There were between three and eight plasmids detected in each isolate (Fig. 2d). Whilst the majority of pairs demonstrated identical plasmid profiles, indicating stable plasmid carriage during the likely GIT–blood transition, Pair 1 demonstrated loss of IncFIB(K) from the faecal isolate and gain of IncN and IncFII(Yp) plasmids in the blood isolate.

### Phenotypic comparison: AMR

**Fig. 3.**
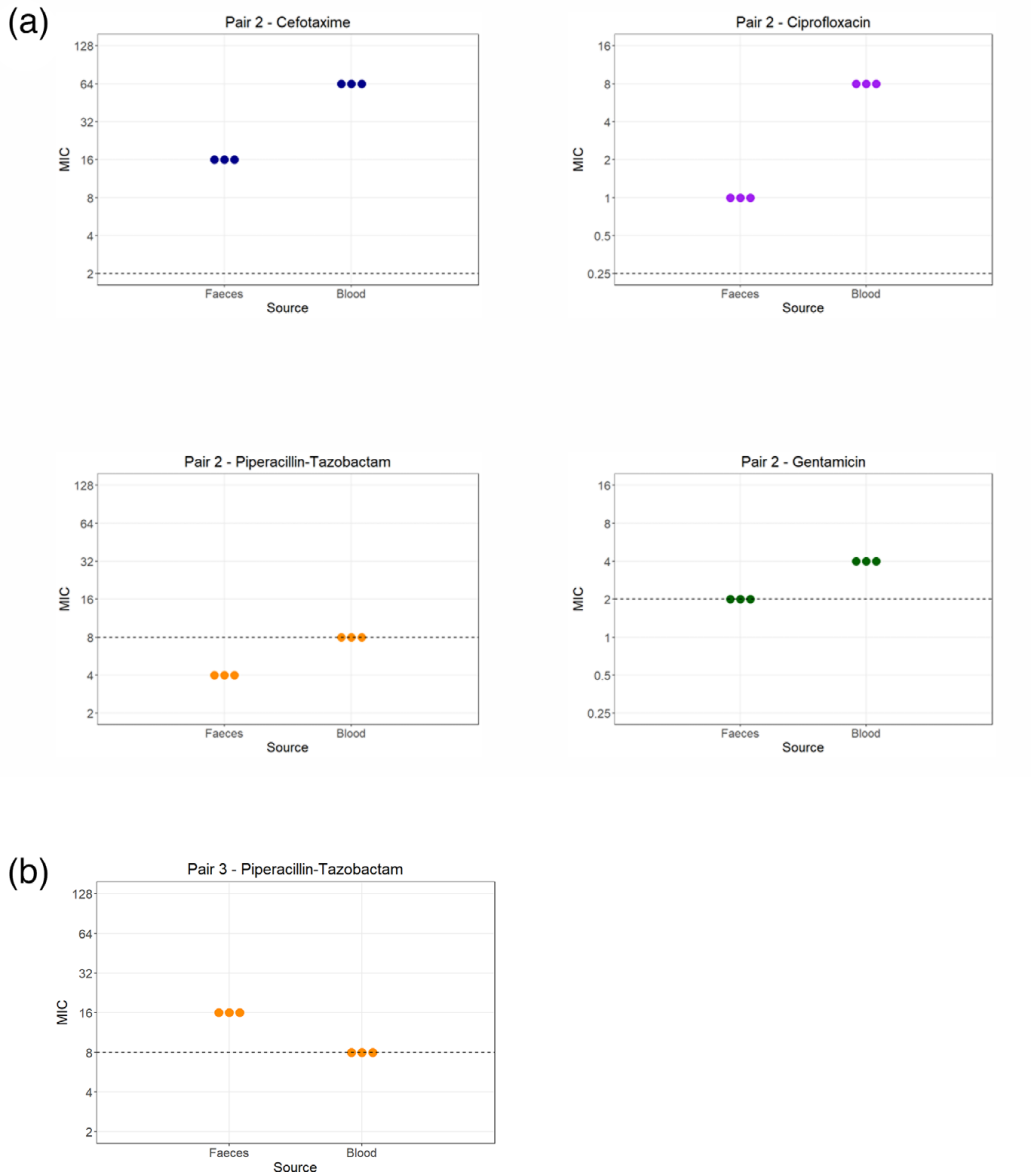
(a) MIC (µg/ml) as determined by broth microdilution of ciprofloxacin, co-amoxiclav, piperacillin-tazobactam, cefotaxime and gentamicin for Pair 2. Each plotted point represents the MIC (µg/ml) of a single biological replicate in broth microdilution. Dashed line=EUCAST clinical breakpoint for MIC. (b) MIC as determined by broth microdilution of piperacillin-tazobactam for Pair 3. Each plotted point represents the MIC of a single biological replicate in broth microdilution. Dashed line=EUCAST clinical breakpoint for MIC.

Disc diffusion testing was used to screen for a change in AMR between faecal and blood isolates of each pair. A change in resistance phenotype was identified for three drug–bug combinations in two out of five pairs (Fig. S1). There was also a statistically significant difference in zone size between faecal and blood isolates that did not cross the EUCAST clinical breakpoint for four drug–isolate combinations in two out of five pairs (Fig. S2 and Table S3). These within-pair differences were only confirmed by broth microdilution in two pairs. Pair 2 demonstrated an increase in MIC for ciprofloxacin, piperacillin-tazobactam, cefotaxime and gentamicin in the blood isolate compared to its faecal counterpart, indicating increased resistance development to these four antimicrobials between the two sampling timepoints (Fig. 3a). In Pair 3, MIC was decreased for piperacillin-tazobactam in the blood isolate compared to faecal, indicating increased susceptibility development in GIT–blood transition (Fig. 3b).

### Phenotypic comparison: biofilm formation

**Fig. 4.**
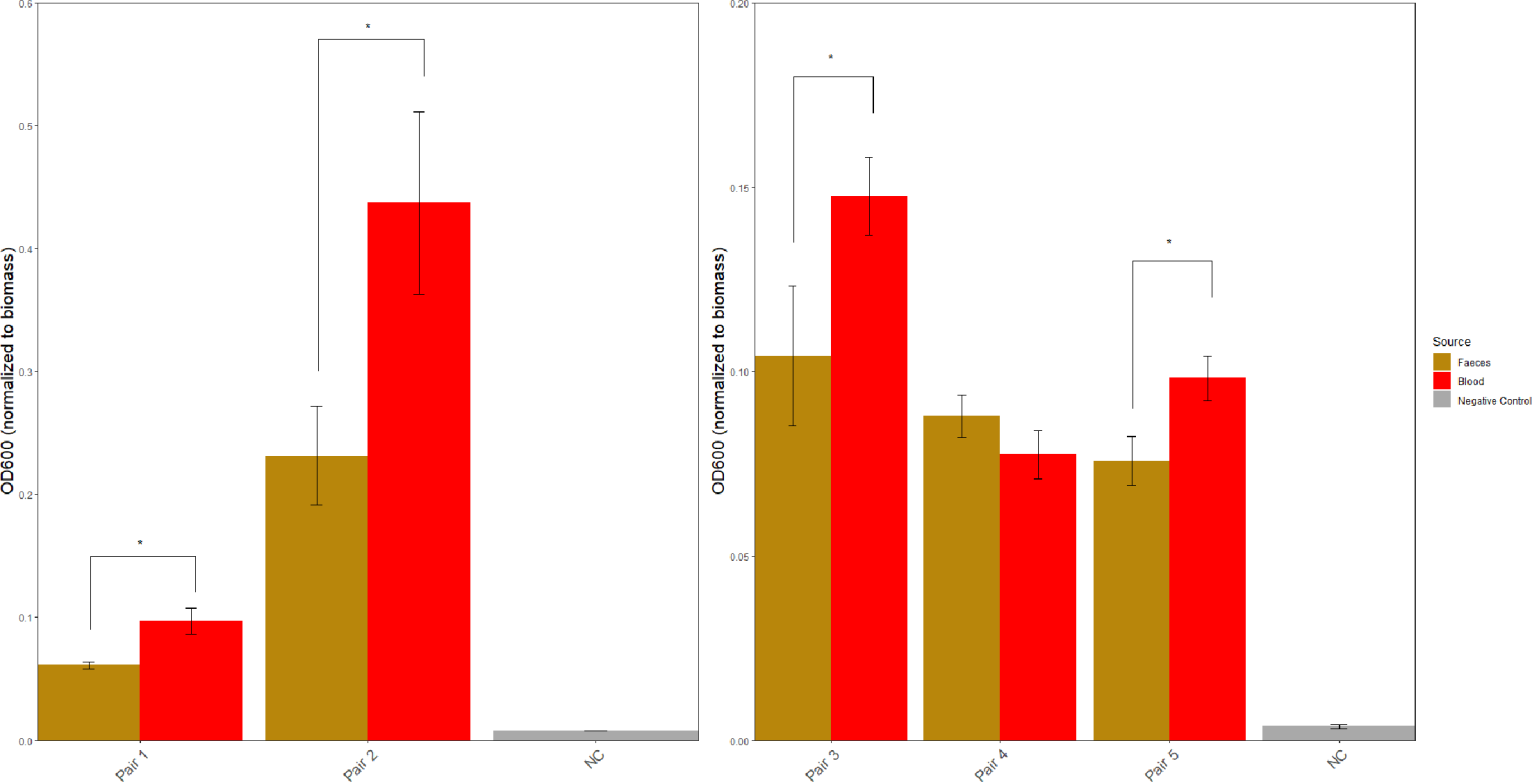
Crystal violet assay for biofilm formation in a 96-well plate. Each bar represents mean OD_600_ (*n*=12) for faecal and blood isolates after crystal violet staining, normalized to biomass. This is used as the measure of biofilm formation capacity. Error bars represent sem. *=statistical significance as determined by paired t-test, *P*=0.05.

Because all patients were found to be diagnosed with CLABSI, and given the importance of biofilm formation in CLABSI pathogenesis, we investigated for any difference in biofilm formation between faecal and blood isolates in each pair. In four out of five pairs, blood isolates demonstrated significantly increased biofilm formation in comparison to the faecal counterpart, as determined by a paired t-test (Fig. 4). All *P*-values are stated in Table S4. Overall, the isolates in Pair 2 demonstrated the strongest biofilm formation.

### Comparative genomics: Breseq analysis

**Fig. 5.**
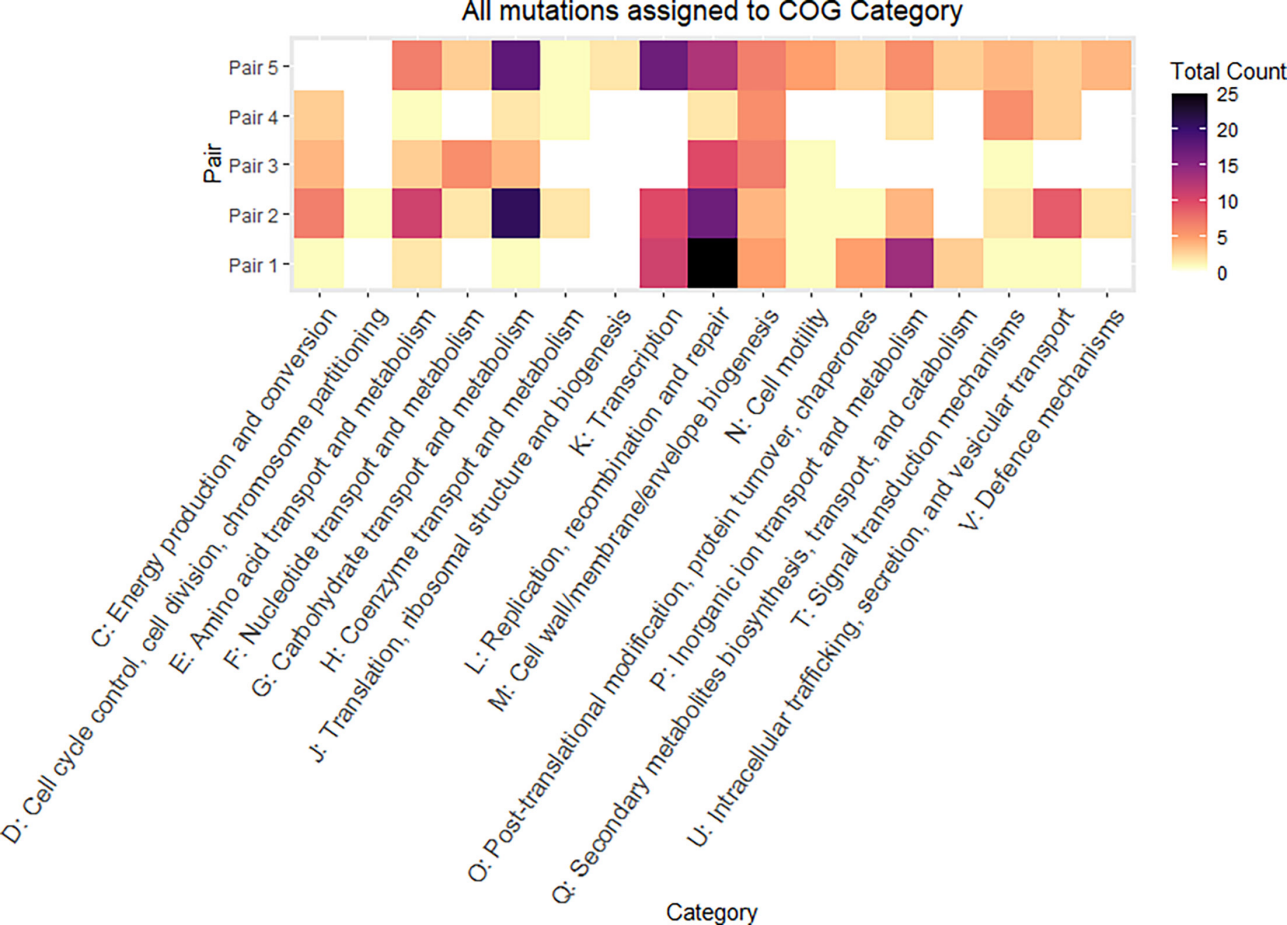
Heatmap of all mutations assigned to COG categories across isolate pairs. Each tile represents the total count of mutations within each COG category for each isolate pair. White tiles represent the absence of mutations in a specific COG category. Mutations observed when comparing blood isolate to corresponding faecal isolate.

Breseq was used to identify mutations in the blood isolate when compared to the faecal isolate. Most mutations were indels, and the majority of these were ‘Function unknown’ or ‘Unclassified’ (Fig. S3 and Table S2). Mutations occurring within 100 bp of genes with a known function were assigned to COG categories (Fig. 5). There were five categories in which mutations occurred in all pairs. Of these, most occurred in ‘L: Replication, recombination and repair’, ‘E: Amino acid transport and metabolism’ and ‘G: Carbohydrate transport and metabolism’.

**Fig. 6.**
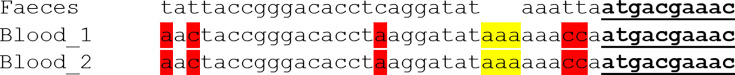
Alignment of upstream intergenic region and first 10 bp (underlined) of *hha* gene of faecal isolate and two copies in blood isolate. (Yellow=duplication of bp. Red=iSNP).

No mutations were identified in specific ARGs identified by ResFinder; however, four pairs demonstrated mutations in genes encoding efflux pumps and transporter proteins involved in AMR (Table 4). Four pairs demonstrated mutations in at least one known virulence factor gene. Two pairs demonstrated mutations in or related to previously reported bloodstream survival factors. All pairs demonstrated a variety of mutations in genes related to biofilm formation. In Pair 1, there was evidence of acquisition of another copy of the *hha* gene, along with modification of the Shine–Dalgarno intergenic region (Fig. 6).

**Table 4.** Specific mutations identified in relation to phenotypes of interest. Mutations observed when comparing blood isolate to corresponding faecal isolate

Pair	Mutation type	Mutation	Annotated gene	Relevance of mutation
1	nsSNP	D47E (GAC→GAA)	*ompk36*	Bloodstream survival factor
	nsSNP	Q62K (CAA→AAA)	*dam*	Bloodstream survival factor
	nsSNP	T57A (ACT→GCT)	*dam*	Bloodstream survival factor
	nsSNP	Q141L (CAG→CTG)	*dam*	Bloodstream survival factor
	Insertion	+GGT	Haemolysin-expression-modulating protein/hypothetical protein	Regulator of virulence factors, known to regulate biofilm formation
	iSNP	2 bp→TT	Haemolysin-expression-modulating protein/hypothetical protein	Regulator of virulence factors, known to regulate biofilm formation
	iSNP	G→T	Haemolysin-expression-modulating protein/hypothetical protein	Regulator of virulence factors, known to regulate biofilm formation
	iSNP	A→G	Haemolysin-expression-modulating protein/hypothetical protein	Regulator of virulence factors, known to regulate biofilm formation
	iSNP	A→T	Haemolysin-expression-modulating protein/hypothetical protein	Regulator of virulence factors, known to regulate biofilm formation
	sSNP	A40A (GCT→GCC)	Haemolysin-expression-modulating protein	Regulator of virulence factors, known to regulate biofilm formation
	sSNP	R11R (CGC→CGG)	Haemolysin-expression-modulating protein	Regulator of virulence factors, known to regulate biofilm formation
	Deletion	Δ916 bp	Fec operon	Iron uptake and utilization
2	nsSNP	T37I (ACT→ATT)	Multiple antibiotic resistance protein MarB	Drug-efflux pump implicated in antibiotic resistance
	Deletion	Δ348 bp	YcaD – MFS transporter	Drug efflux pump implicated in antibiotic resistance
	Deletion	Δ254 bp	Permease of drug/metabolite transporter	Drug efflux pump implicated in antibiotic resistance
	Deletion	Δ250 bp	Inner membrane protein/drug antiporter	Drug efflux pump implicated in antibiotic resistance
	Deletion	Δ243 bp	LysR	Transcriptional regulator implicated in antibiotic resistance
	Deletion	Δ241 bp	MFS-type transporter	Drug efflux pump implicated in antibiotic resistance
	Deletion	Δ239 bp	RND efflux transporter	Drug efflux pump implicated in antibiotic resistance
	Deletion	Δ236 bp	LysR transcriptional regulator	Drug efflux pump implicated in antibiotic resistance
	Deletion	Δ321 bp	*acrR*	Transcriptional regulator implicated in antibiotic resistance and biofilm formation
	nsSNP	A434S (CCC→TCC)	Protease III precursor (EC 3.4.24.55)	Biofilm formation
	nsSNP	V701A (GTG→GCG)	T6SS component TssM (IcmF/VasK)	Virulence factor, involved in biofilm formation
3	Deletion	Δ235 bp	Lipopolysaccharide ABC transporter	Transporter protein implicated in antibiotic resistance
	nsSNP	T115A (ACC→GCC)	Phage endopeptidase Rz	Phage protein, adjacent to virulence factor *iss* (bloodstream survival)
	nsSNP	L237P (CTT→CCT)	Mobile element protein	MGE adjacent to Fec operon (iron uptake and utilization)
	nsSNP	A238G (GCC→GGC)	Mobile element protein	MGE adjacent to Fec operon (iron uptake and utilization)
	nsSNP	S932T (AGC→ACC)	Antigen 43	Virulence factor, involved in biofilm formation
	nsSNP	A763V (GCC→GTC)	Antigen 43	Virulence factor, involved in biofilm formation
	sSNP	G765G (GGA→GGT)	Antigen 43	Virulence factor, involved in biofilm formation
4	Deletion	Δ286 bp	MFS type transporter	Drug efflux pump implicated in antibiotic resistance
	sSNP	G977G (GGG→GGT)	Antigen 43	Virulence factor, involved in biofilm formation
	sSNP	G972G (GGT→GGA)	Antigen 43	Virulence factor, involved in biofilm formation
	nsSNP	S932T (AGC→ACC)	Antigen 43	Virulence factor, involved in biofilm formation
	nsSNP	V375A (GTC→GCC)	Antigen 43	Virulence factor, involved in biofilm formation
	Deletion	Δ265 bp	Ferrochelatase	Iron uptake and utilization
	Deletion	Δ236 bp	Ferredoxin reductase	Iron uptake and utilization
	Deletion	Δ119 bp	cog3210	Iron uptake and utilization
	Deletion	Δ95 bp	cog3210	Iron uptake and utilization
5	sSNP	L123L (CTG→TTG)	Manganese ABC transporter, inner membrane permease protein SitC	Iron uptake and utilization
	Deletion	Δ297 bp	Transcriptional regulator, *acrR* family	Transcriptional regulator implicated in antibiotic resistance and biofilm formation
	Deletion	Δ295 bp	Spermidine/putrescine ABC transporter	Transporter protein implicated in antibiotic resistance
	Deletion	Δ281 bp	Fe^2+^ ABC transporter, substrate-binding protein	Iron uptake/homeostasis
	nsSNP	V32E (GTG→GAG)	Transcriptional repressor of aga operon	Transcriptional regulator involved in biofilm formation
	nsSNP	M300L (ATG→CTG)	Lipopolysaccharide export system permease protein LptG	Biofilm formation
	Deletion	Δ246 bp	TctA transporter	Drug-efflux pump implicated in antibiotic resistance
	Deletion	Δ245 bp	AaeAB efflux system	Drug efflux pump implicated in antibiotic resistance

## Discussion

In this study, we compared blood isolates with the previously acquired faecal isolates from five children with GNBSI and, in each instance, demonstrated concordance in sequence type and species-specific typing methods. This is highly suggestive that the faecal–blood isolate pairs were genomically ‘linked’ and that the invasive blood isolate was likely to have been acquired from the GIT. ANI has been used as a marker of genomic relatedness in previous studies comparing faecal–blood isolates in children [24,25], but there is no defined consensus on a threshold that indicates isolates as similar enough to be linked. Thresholds of 99.99% have been previously used to indicate isolates being the same strain [26]. Using this threshold, four out of five pairs (Pairs 2–5) shared ANI values convincing of being the same strain. The lower ANI in Pair 1 (99.82%) could be somewhat explained by the identified plasmid changes in this pair. This is consistent with observations that MGE movement can lead to a lower ANI in isolates that are highly likely to be concordant, as noted by Vornhagen *et al*. when comparing wzi-type concordant rectal and blood *K. pneumoniae* isolates [27]. However, this finding could also be indicative of the blood isolate having evolved to be a different clade of the same sequence type as the faecal isolate in this pair, as indicated by the higher number of core genome SNPs calculated in further analysis.

The number of core genome SNPs between faecal and blood isolates in each pair ranged from 38 SNPs (Pair 2) to 318 SNPs (Pair 1), indicating varying degrees of evolutionary distance between faecal and blood isolates in each patient. It is possible that our BSI isolates, although related, are not immediate descendants of the cognate faecal isolate and that there could have been closer ancestors in the GIT which were not picked up during sampling. Similarly, there may have been evolution within the gut and the blood, giving rise to multiple descendants, of which we isolated one. A previous study that identified concordant faecal and blood isolates in patients with *E. coli* bloodstream infections noted that the number of core genome SNPs between faecal and blood isolates was 0 [28]. Another study comparing faecal and blood *K. pneumoniae* isolates found a core genome SNP difference of 6 SNPs between isolates considered to be concordant strains in 1 patient and 76 SNPs between concordant isolates from another patient [29]. In our study, the number of SNPs we found is generally higher and was not proportional to the number of days between faecal and blood isolation. This may represent varied and convoluted routes taken from the GIT to the blood, e.g. via other body tissues in each case of GNBSI, requiring adaptation to multiple niches – as opposed to direct GIT–blood translocation.

Alternatively, the higher number of core genome SNPs in Pair 1 could be considered to contradict the hypothesis that this represents a ‘linked’ pair. However, as with ANI, there is no SNP threshold that is universally agreed as defining isolates as ‘linked’. For example, whilst 1 study used a threshold of 17 core genome SNPs for this, another study looking at persistence of *E. coli* isolates between GI and urinary tracts used a threshold of ≤500 core genome SNPs [28,30]. These differences could also be due to differences in sequencing and bioinformatic technologies, as well as the reference genomes used in different studies. Such differences in methodology are recognized as a key barrier to the determination of a defined threshold for strain similarity [31,33]. In addition, the expected rate of mutation of isolates moving between different body niches is currently unknown and unpredictable [34], adding to this challenge. When comparing isolates from the two different patients with *K. pneumoniae* infection, there are over 30,000 core genome SNPs between each. A similar number is seen when comparing the *E. coli* isolates of Patient 5 to both Patient 3 and Patient 4. However, the number of core genome SNPs between Patients 3 and 4 is more comparable to those seen within the pair of Patient 1. This raises suspicion of a common source of colonization of these patients within the intensive care unit during the time period the isolates were taken. However, granular data of overlapping hospital admission times are unavailable.

ARG profile and, subsequently, AMR phenotypes were compared within ‘linked’ pairs. The transition from GIT colonization to blood invasion was associated with acquisition of an ARG in one out of five pairs (Pair 4) – and in this case, the acquisition of *bla*TEM-1B did not lead to increased resistance to any of the beta-lactam/inhibitor combinations tested. Only in one pair (Pair 2) did we observe that increases in resistance to any antimicrobials occurred between the two sample timepoints. This is despite the fact that in all five cases, the patient from whom isolates were obtained received multiple antimicrobials during hospital admission. In contrast, for Pair 3, increased susceptibility to piperacillin-tazobactam was noted. These findings may reflect varying selection pressure exerted by dose and duration of antimicrobial therapy, in balance with environmental adaptation. Unfortunately, the study is limited by the lack of granular data on antimicrobial exposure. These phenotypic changes in AMR are not explained by acquisition or loss of ARGs (Fig. 2a). However, by using Breseq for whole-genome comparison, we identified deletion of drug-efflux pump repressors that could putatively explain this observation. Several drug-efflux pumps are conserved across Gram-negative species and implicated in AMR. These include MFS pumps, RND pumps and ABC transporters. The *acrAB-tolC* efflux pump is an RND pump implicated in resistance to multiple antimicrobials [35]. It has multiple regulatory pathways, including the *acrRAB* operon, in which *acrR* is a repressor, and the *marAB* operon, with *marB* as a repressor. In Pair 2, an nsSNP in *marB*, along with deletion of *acrR*, was identified (Table 4), which could lead to increased efflux pump activity, explaining the changes in susceptibility. The deletion of *acrR* is particularly interesting because a previous study reported that *acrR* knockout in *K. pneumoniae* isolates resulted in increased MIC to the same antimicrobial classes as seen here [36]. Previous literature also reports an association between *acrR* disruption and a multi-drug resistant phenotype [37,38]. However, it must be noted that an *acrR* deletion was identified in Pair 5 but without a change in susceptibility, possibly explained by the variation in other efflux pump changes seen in each pair.

In five out of five cases in this study, GIT–blood transition was not associated with the acquisition of virulence genes when comparing blood isolates to the presumed faecal ancestor. Because all cases of GNBSI in this study were cases of CLABSI, biofilm formation was chosen as a virulence phenotype of interest. The finding that four out of five blood isolates exhibited an increase in biofilm formation compared to faecal counterparts suggests that in-host adaptation towards increased biofilm formation may be implicated in the pathogenesis. As noted, the observed phenotypic changes are not explained by acquisition of virulence genes involved in biofilm formation, but comparative genomic analysis using Breseq again identified a variety of putative mechanisms that could potentially underpin our phenotypic observation.

In Pair 2, the *acrR* deletion explored above could also be responsible for increased biofilm formation, as *acrAB-tolC* has a central role in biofilm formation in *E. coli* and *K. pneumoniae* [39,40]. Studies also link biofilm formation, AMR and *acrAB-tolC* activity. In a comparison of *K. pneumoniae* urinary isolates, Vuotto *et al*. observed that those with the highest biofilm-forming capacity also demonstrated the most AMR, correlating with increased *acrAB* activity [41]. Although only a sample of three isolates, this strikingly mirrors the findings of the *K. pneumoniae* blood isolate of Pair 2, raising the possibility of a single intra-host adaptation contributing to both AMR and biofilm formation. Hennequin *et al*. demonstrated this *in vitro*, reporting that sub-MIC cephalosporin exposure in *E. coli* isolates resulted in enhanced *acrAB* expression and biofilm formation [42]. This could be an example of antimicrobial exposure selecting for a resistance-conferring mutation that also increases virulence and propensity to invasive infection. If seen consistently in a larger cohort, this could suggest a promising interventional target. For example, RND efflux pump inhibitors have been explored for use as antimicrobial-adjuvant therapy for resistant infections, and for biofilm disruption in invasive infection [43].

Furthermore, in this pair, nsSNPs in two biofilm-related virulence genes, *IcmF* and protease III precursor, were identified, possibly contributing to the phenotypic change. Similarly, in Pair 3, two nsSNPs in the biofilm-associated virulence gene ‘antigen 43’ were noted. Mutations in this gene were noted when Nielsen *et al*. compared ‘linked’ urine and faecal isolates and hypothesized this related to increased biofilm formation [44].

Pair 1 exhibited a potential link between biofilm formation and plasmid acquisition. This pair underwent loss of IncFIB(K) and acquisition of IncFII(Yp) between faecal and blood isolates (Fig. 2d). Breseq analysis revealed a duplication of the *hha* gene in the blood isolate compared to the faecal isolate (Table 4), with the additional copy residing on a contig containing multiple IncF-related genes, suggesting plasmid-mediated acquisition. The *hha* gene encodes a haemolysin-expression-modulating protein and is a regulator of virulence factors involved in cell adhesion and biofilm formation in Gram-negative species. Studies in *E. coli* have identified *hha* as a positive regulator of biofilm formation and of virulence [45,46]. This is less studied in *K. pneumoniae*; however, Bandeira *et al*. described *hha* presence to be associated with *K. pneumoniae* biofilm-forming isolates, particularly on indwelling central lines [47]. Plasmid-mediated acquisition of *hha* was described by Krall *et al.* who compared ‘linked’ faecal and blood isolates, finding that *hha* acquisition on an IncFII plasmid increased the invasiveness of *E. coli* isolates in a gut-organoid model [48]. They concluded that *hha* was involved in GIT–blood adaptation but did not explore biofilm formation specifically. Here, one could similarly hypothesize that IncFII plasmid-mediated acquisition of *hha* could be an adaptive change leading to increased biofilm formation and procession to GNBSI. Other studies report that *hha* has a negative influence on biofilm formation [49,50], which this hypothesis contradicts. Interestingly, in both copies of the *hha* gene in the blood isolate, a 3 bp GGT insertion was noted 1 bp downstream of *hha*. Upon gene alignment, this appeared to be an AAA duplication, disrupting the Shine–Dalgarno sequence 7 bp before the gene (Fig. 6). This could potentially reduce the efficiency of protein translation. Hence, one could equally hypothesize, assuming a negative-regulatory role of *hha*, that after plasmid acquisition during GIT colonization, the compensatory mutation in the Shine–Dalgarno region led to increased biofilm formation and aided bloodstream invasion. Further investigation is required to clarify the role of *hha* in invasion and the combined effect of the observed SNPs, Shine–Dalgarno mutation and *hha* copy number on biofilm formation.

The transition from GIT to blood and subsequent survival in the bloodstream will involve the ability to adapt to a harsh and dynamic environment, with factors such as varying nutrient abundance and host immunity to contend with. As noted, this adaptation did not appear to result from acquisition of virulence genes in these five cases but was associated with varied changes across the genome. No single specific mutation occurred in all pairs, likely reflecting the differing clinical scenarios and selection pressures driving diverse evolutionary trajectories en route to bloodstream infection. We did find a small number of common mutations within the *E. coli* strains and across two of the three *K. pneumoniae* strains (Table S2); however, in all pairs, most mutations occurred in the COG category 'Replication, recombination and repair', including a mutation in *dam*, a known bloodstream survival factor, in Pair 1 [51]. These genes are involved in environmental and stress response, and selection for mutations could be expected in adaptation to challenging environments, for example, maintaining efficient DNA repair mechanisms in the presence of reactive oxygen species, host immune defences or nutrient variability. Another commonality was mutations in 'cell wall/membrane/envelope biogenesis', 'cell motility' and 'signal transduction' categories. Such mutations might confer altered chemotaxis or adherence, allowing tissue invasion. Few mutations were observed in 'Cell cycle control and repair' and 'Translation, ribosomal structure and biogenesis'. This demonstrates the strong selective pressure to conserve key cellular processes integral to bacterial survival during the transition between GIT and blood environments. Many mutations were classified as ‘Function Unknown’ within COG categories but may hold significance. For example, in Pair 3, an nsSNP was identified in a phage endopeptidase gene adjacent to the gene *iss*. The *iss* gene is associated with bloodstream survival in *E. coli* through mediating serum and complement resistance, and increased mRNA copy number has been associated with increased serum survival [52,53]. Mutations in the adjacent phage endopeptidase could influence the activation or expression of *iss*, with effects on bloodstream survival. This represents a potential avenue for further research.

When comparing blood isolates to their faecal ancestors using Breseq, we noted mutations related to carbohydrate and amino acid transport in five out of five pairs. This included an nsSNP in *ompk36* in Pair 1, a transport protein also previously identified as a bloodstream survival factor in *K. pneumoniae* [54]. Further, we identified mutations related to iron uptake utilization in four out of five pairs. Iron availability and chemical form vary in different tissues, and a ‘functional hierarchy’, whereby iron uptake systems are utilized variably across environments, has been discussed by Garcia *et al.* in the context of urinary tract infection [55]. The findings of *fec* operon deletion in Pair 1 and the transposase mutation near the fec operon in Pair 3 may suggest a genomic shift away from ferric citrate uptake, whilst the *sitC* virulence gene mutation and ferrous (Fe^2+^) ABC transporter deletion in Pair 5 may suggest a shift away from Fe^2+^ uptake. These mutations likely reflect adaptation towards iron acquisition in the bloodstream, where ferric citrate is less abundant than the gut and Fe^2+^ is less abundant due to oxygenated conditions. Further exploration could inform therapeutic strategies. For example, *tonB*, which regulates the *fec* operon, has been explored as a potential drug target against *E. coli* [56]. However, this may not be an appropriate target in the context of bloodstream infection if functional redundancy of the *fec* operon is a common adaptation.

The strengths of this study include the combination of genomic with phenotypic comparison, using isolates from the same patient to gain insight into within-host evolution and link phenotypic differences to changes at the genomic level. To our knowledge, there are few studies in the existing literature that have investigated this. Further, we compared mutations across the whole genome, rather than focusing only on known ARGs and virulence genes, identifying potentially relevant mutations that would have been missed by a narrower approach. Limitations of our study lie in the fact that it cannot be definitively concluded that these pairs are ‘linked’, given the lack of an existing robustly defined threshold for strain similarity/genomic relatedness – this, in itself, highlights an area for further work. In addition, the retrospective nature and ability to only include five cases of GNBSI, without granular clinical data, mean the significance and generalizability of these findings cannot be assumed. The use of short-read rather than long-read sequencing makes it harder to discern plasmid-mediated changes that could contribute to adaptation. Further, phenotypic assays for biofilm formation demonstrated changes *in vitro* but likely do not reflect true clinical conditions. Whilst this study shows individual instances of mutations of potential clinical relevance in each pair which may represent avenues for further study, it is clear that there is a diverse range of possible evolutionary trajectories in GIT–blood transition, even in this small study, so a common pathway for clinical intervention may be difficult to elucidate. Future study with a larger sample size that used faecal isolates from proven ‘linked’ pairs to colonizing isolates that did not achieve bloodstream invasion could be a more promising avenue to identify pathogen factors, e.g. virulence gene SNPs that may predispose to bloodstream infection. Previous case-control studies with this approach have been used, but not including WGS data [57]. Comparative analysis, including isolates from other body sites, such as the urinary and respiratory tracts, would also be beneficial. This, along with information around antimicrobial exposure, clinical severity and GNBSI source, would give a more comprehensive insight into the relevance of adaptations to differing clinical scenarios and how findings could translate to clinical intervention.

Overall, this study used genomic and phenotypic comparison of ‘linked’ pairs of blood and faecal *K. pneumoniae* and *E. coli* isolates to investigate evolutionary and adaptive changes relevant to the transition from GIT colonization to bloodstream invasion and GNBSI. Phenotypic changes in AMR were variable; however, biofilm formation and metabolic flexibility were common phenotypic adaptations towards bloodstream infection in this small sample. These changes were not found to be associated with acquisition of ARGs or virulence genes, but rather, with a diverse array of genomic changes, including mutation of virulence genes, efflux pumps and acquisition of plasmid-mediated genes. A small number of common mutations were found between the *E. coli* strains and between two of the three *K. pneumoniae* strains; however, changes in functional categories of genes involved in transport and metabolism were common across all five pairs. These findings demonstrate a snapshot of the vast evolutionary landscape of intra-host adaptation that is traversed in GNBSI pathogenesis. However, significant further study is needed to assess whether these findings could feasibly translate into clinical intervention for GNBSI treatment or prevention.

## Supplementary material

10.1099/jmm.0.002147 Uncited Supplementary Material 1.

10.1099/jmm.0.002147 Uncited Supplementary Material 2.

## Abbreviations

AMR: antimicrobial resistance
ANI: average nucleotide identity
ARGs: antimicrobial resistance genes
AST: antimicrobial susceptibility testing
cgMLST: core genome multilocus sequence typing
CLABSIs: central line-associated bloodstream infections
COG: cluster of orthologous groups
ESBL: extended-spectrum beta-lactamase
GIT: gastrointestinal tract
GNBSIs: Gram-negative bloodstream infections
indels: insertions/deletions
iSNPs: intergenic SNPs
LB: Luria-Bertani
MHB2: Mueller–Hinton broth 2
nsSNPs: non-synonymous SNPs
SNP: single nucleotide polymorphism
sSNPs: synonymous SNPs
WGS: whole-genome sequencing
