# Supplementary material for: From colonization to invasion: genomic and phenotypic comparison of faecal and bloodstream isolates from the same patients
Source: J Med Microbiol. 2026 Apr 8;75(4):002147. doi: 10.1099/jmm.0.002147 (PMC13061263; doi:10.1099/jmm.0.002147)
Supplement: Uncited Supplementary Material 1. [file jmm-75-02147-s001.pdf]

Supplementary figures and tables :

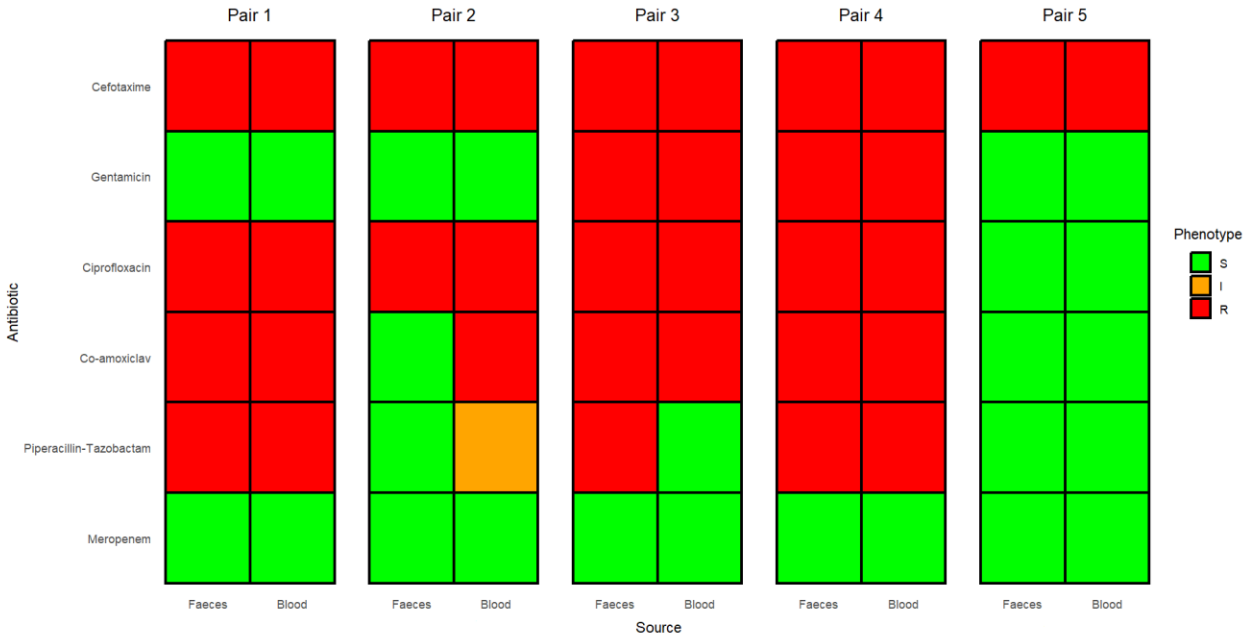

Supplementary figure 1: Mode antimicrobial susceptibility phenotype using disc diffusion testing (n=9) against six antimicrobials, as per EUCAST clinical breakpoints. S=Sensitive, I=Sensitive with increased dosing, R=Resistant.

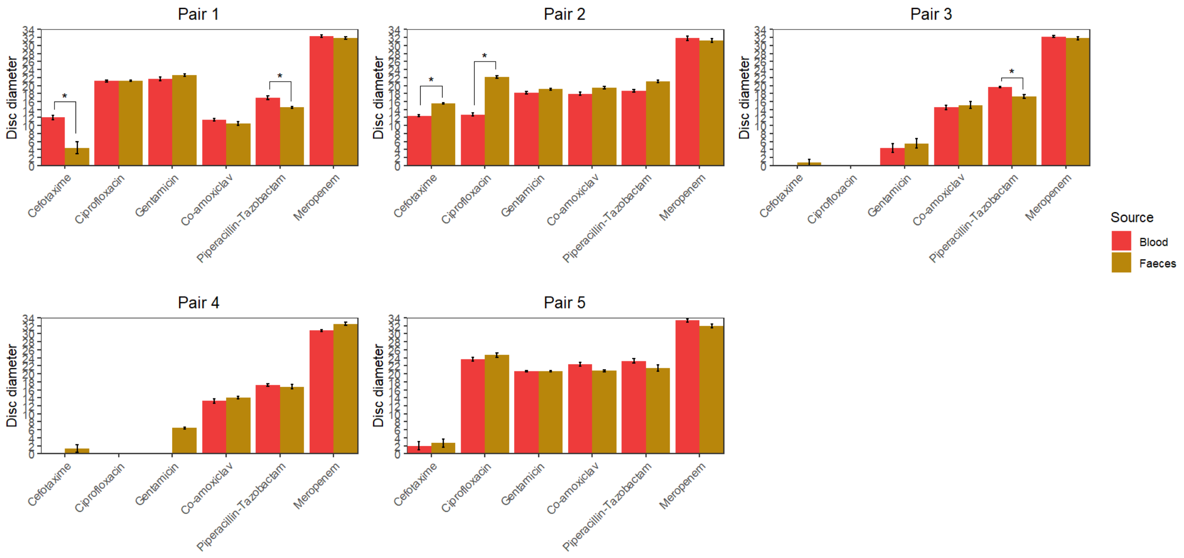

Supplementary figure 2: Mean disc diameter (n=9) of faecal and blood isolates tested against six antimicrobials for all 5 pairs. On each chart, each bar represents the disc diameter of faecal and blood isolates against each antimicrobial. Error bars represent standard error of the mean. * = statistical significance as determined by Mann-Whitney U test, p=0.05.

Supplementary Table 1: Whole Genome Sequencing statistics

Isolate	Total bp in trimmed reads	Number of trimmed reads	N50 of assembled genome	L50 of assembled genome	Coverage (X)	Total bp in assembled genome	Number of contigs	GC Content %
**Faeces 1**	210743741	1412138	99383	19	38	5478748	316	57.3
**Blood 1**	371491090	2488464	103450	19	68	5440837	233	57.3
**Faeces 2**	3047930900	20437228	135138	15	500	6093121	509	56.5
**Blood 2**	977142638	6553008	125383	16	162	6014008	419	56.5
**Faeces 3**	884027184	5919548	159977	11	165	5353515	304	50.7
**Blood 3**	2039292116	13658812	164877	12	380	5360978	337	50.7
**Faeces 4**	1038239903	6964806	157274	12	196	5286737	273	50.6
**Blood 4**	915916316	6142772	138979	13	170	5388799	285	50.6
**Faeces 5**	658599502	4417470	174067	10	122	5393683	445	50.3
**Blood 5**	1251284036	8389470	163504	11	233	5357807	320	50.3

Supplementary Table 2 – see accompanying Excel file.

Supplementary Table 3: Mann-Whitney U Test to ascertain significant difference between faecal and blood isolate disc diameters.

	Pair 1	Pair 2	Pair 3	Pair 4	Pair 5
**Antibiotic**	**P value**				
**Cefotaxime**	0.0008978249	0.0002533103	0.374062797	0.1685887716	0.676193126
**Gentamicin**	0.1108027031	0.0490272876	0.431709250	0.1152119000	1.000000000
**Ciprofloxacin**	0.8385489076	0.0003633622	NA	NA	0.194295441
**Co-amoxiclav**	0.0857751742	0.0162110595	0.926173749	0.2554864522	0.005570945
**Piperacillin-Tazobactam**	0.0011324664	0.0014335714	0.000565947	0.6484983709	0.104526039
**Meropenem**	0.3025207858	0.3866889310	0.230473319	0.0563107490	0.064250082

Supplementary table 4: Paired T test to ascertain significant difference of mean OD600 between faecal and isolate pairs.

Pair	P value
1	0.011829607
2	0.002514331
3	0.045537335
4	0.183078015
5	0.022019967

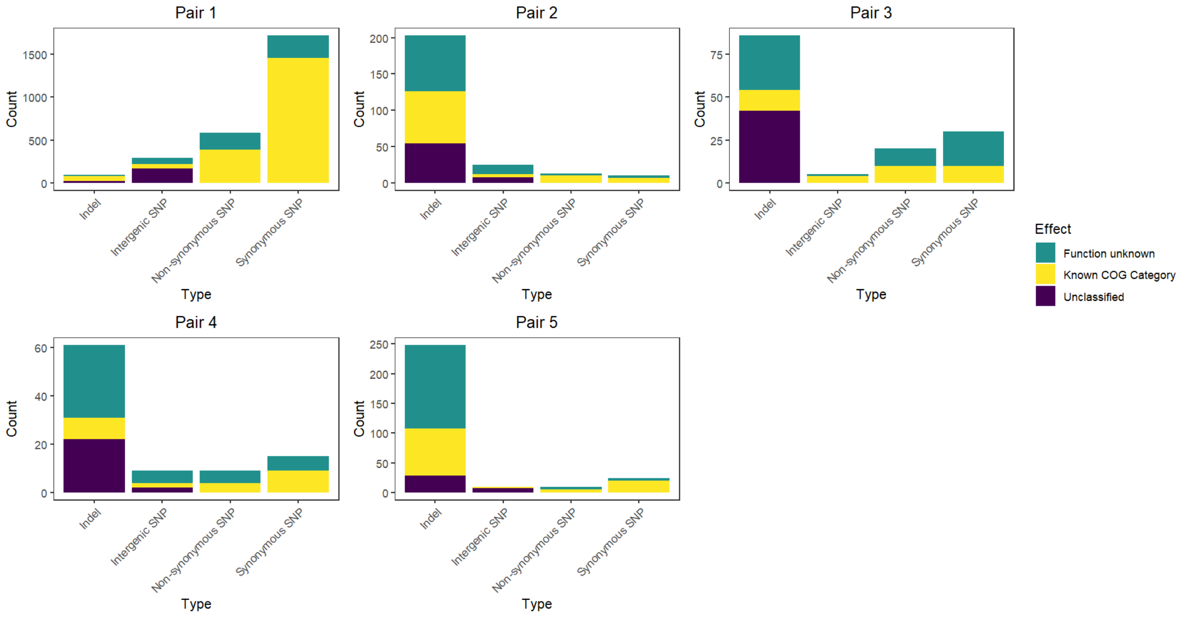

Supplementary figure 3: Mutation type in each isolate pair. On each chart, each bar represents the number of each type of mutation identified by Breseq (Indel, Intergenic SNP, Non-synonymous SNP, and Synonymous SNP) categorised by where mutation occurred. Function unknown/known COG category = mutation occurred in or within 100bp of a gene of either unknown function or known function respectively. Unclassified = mutation did not occur within 100bp of a gene.
